# Testing the Constraint Theory of Addiction: Cannabis Constraints Discriminate Users from Nonusers and Heavy from Light Users

**DOI:** 10.1155/2020/3427270

**Published:** 2020-05-23

**Authors:** Richard Hammersley, Nick Holmes, Marie Reid

**Affiliations:** Department of Psychology, Faculty of Health Sciences, University of Hull, Hull, UK

## Abstract

Constraint theory (Hammersley, 2014) offers a novel way of understanding addiction as a lack of cognitive, behavioural, and social constraints on substance use. Here, cannabis constraints were studied in a large online opportunity sample: *N* = 302; 205 men, 97 women. Age ranged from 14 to 60 years (mean = 25, SD = 8.0). Most participants were from UK or North America. Participants completed a questionnaire assessing 15 cannabis constraints and standard self-report frequency measures of drug use. Factor analysis of the constraint questionnaire found 15 factors, similar to those proposed theoretically. These factors could discriminate well between past and current users and heavy and light users. The best discriminator was concerns about the possibility of becoming addicted; the less concerned the heavier was use, although those who actually felt addicted were more concerned than others. Past users also constrained due to using legal highs instead, concerns about illegality, and using only when others used. Light users constrained due to availability and cost issues, as well as unpleasant effects. These findings suggest that there is utility in constraint theory and that heavy use occurs due to a relative lack of constraints.

## 1. Introduction

The brain disease model of addiction (BDMA) remains dominant in theorising about the causes and life course of substance use and addiction, despite repeated criticisms of this paradigm [1–7]. Characterising it as a paradigm is more appropriate because addiction cannot be defined independently of its paradigm; the conceptual and philosophical approach to a scientific problem is influenced by the cultural and other implicit assumptions of the scientists [8]. However, a “model” commonly means a representation of something that shares important characteristics with that thing. Models can only be made within a paradigm, although in science practice models, theories and paradigms are rarely well distinguished.

BDMA models have known weaknesses (see above references), but it remains tenaciously influential, perhaps in part because a coherent alternative paradigm is lacking. Hammersley [9] proposed the constraint theory of addiction as an alternative, where addiction is caused by a lack of constraints on substance use, rather than by the specific effects of drugs on brain or behaviour. The specific effects of drugs occur whenever drugs are used, whether by an “addict” or another type of user, but do not cause addiction; they are necessary but not sufficient causes. Rather, enjoyable drug plus a relative lack of cognitive, affective, or social constraints on use tends to lead to addiction, problems, or a substance use disorder. Constraint theory [9] is a model of drug use, but there is also an implicit paradigm in the paper.

In this paradigm, substance use is not caused, it is moderation or avoidance of substance use that is caused, by the existence of constraints on use. Put in another way, if a pleasurable drug is available to use and there are no consequences (constraints) of use, then people will use it. Within the paradigm, this could apply to any highly pleasurable activity, as will be illustrated shortly. Yet, few people, even “addicts,” have no constraints at all on use. For example, drug injectors describe taking precautions to avoid injecting in front of their children [10, 11].

The constraints that currently exist and which of them are applicable to an individual substance user are the matters to be established empirically; they will vary according to personal, social, and cultural circumstances. For example, in the heyday of cigarette smoking in the UK in the mid-1970s, there were very few constraints on use at all. There were ashtrays in cinemas and university seminar rooms. Cigarettes could be bought from vending machines in the street. The local tobacconist and sweet shop sold single cigarettes. With few constraints, in 1974, over 40% of adult women and over 50% of men smoked cigarettes at the time that they were surveyed [12]. Of cigarette smokers, over 30% of women and over 50% of men smoked at least 20 a day.

The dramatic decline in tobacco smoking in the years since has occurred due to incremental increases in the financial, legal, interpersonal, cultural, and legal constraints on smoking, driven by widespread acceptance of the very serious health consequences of smoking. A BDMA model of nicotine addiction would have to predict that most or all of this decline was due to people not starting to smoke in the first place, so that their neuropsychopharmacology could not be altered by nicotine, but a pseudocohort analysis of the same data shows that as each cohort aged a higher proportion became nonsmokers [12], for as constraints increased, more people stopped. Indeed, there is a large literature on using factors such as price, availability, and norms to attempt to control substance use.

This contrasts with the core supply reduction approach to illicit drug use, which reduces or eliminates the tactical regulation and management of other constraints on use. How should cannabis be packaged? Where should it be sold? How should products be priced? And so on. Media reports from places where the legal status of cannabis has changed do not suggest that emerging practices are evidence-based or even well considered. There is a developing need to understand cannabis constraints as cannabis use becomes endemic, more widely sold for medical and recreational use, and use pattern change [13]. Additionally, concerns remain that cannabis use may be linked to psychosis [14, 15] and depression [16], as well as cognitive deficits, mood disorders, and substance use problems [17].

As previously discussed [9], constraint theory's closest theoretical relative is the extended theory of planned behaviour (TPB) [18, 19]. However, TPB is about planned behaviour that originates with an intention, whereas not all substance use or other habitual behaviour is planned, and when habits are strong, then intentions may be weak or nonexistent [20]. Constraints in principle do not require intentions, so if and when constraints are implemented via intentions are a matter to be determined empirically. Constraints can also be nonverbal habits of which the person may not be fully aware. For example, most people who do not drink alcohol at work or first thing in the morning do not require forming an intention or even thinking about it. One's morning routines, habits, or schemata simply do not contain alcohol as a possibility. On the other hand, unrepentant heavy drinkers may have to decide whether or not to have an eyeopener.

Turning to a cannabis example, an occasional user of cannabis may be offered a spliff at a social event. Depending on the nature of the event and a variety of other constraints, they may take a toke or not. Moreover, in theory, they might toke despite having a prior intention to avoid cannabis in future, because there were few constraints on doing so (they are at a music festival surrounded by happy cannabis smokers whom they will never see again, so social constraints are weak), or they might decline to toke despite having a prior intention to use cannabis next time they were offered it (their boss is covertly offering her cannabis in their offices, so rules about workplace comportment and concerns about entrapment constrain them). They do not have to consciously process all this to be constrained.

In TPB, various psychological factors predict intentions, which in turn predict behaviour. In constraint theory, substance use is not driven by a collection of psychological factors but rather tends to occur in the absence of constraints. Consequently, in some conditions, such as music festivals, even people very disinclined to use cannabis may actually use because they are away from their families and everyday lives, including everyday constraints on their use. However, like TPB, the current version of constraint theory is more oriented towards predicting trends in general behaviour than about predicting specific behaviour episodes. This is partly because, as with social cognition in general, it is challenging to study cognitions about substance use episodes as they happen. It is easier to look at generic trends, as in this paper.

Hammersley [9] identified 15 general types of constraints: religious or moral beliefs; jaded regarding consumerism; family and friends opposed to use; limited opportunities for use; other things to do; lack of friends to use with; no effects of substance; disliked effects; lack of stress; like the drug too much; health scare or problems; recognise immanent addiction or dependence; legal risks; lack of functional availability; too expensive. These were established empirically by consulting experts, but it remains to be established (a) whether these constraints exist amongst substance users and (b) to what extent they predict substance using behaviour, including self-reported behaviour. Here, the term “addiction” is used in the everyday sense whereby people report being “addicted” or concerned about “addiction” to distinguish it from the diagnostic terms substance use disorder, misuse, and dependence.

The study reported here was designed to examine the factor structure of cannabis use constraints, then to test the basic prediction that lack of constraints should predict the extent of substance use, and the severity of self-reported addiction, along with a more nuanced test regarding which constraints are the best predictors of substance use. For, there are no a priori reasons to assume that all 15 constraints are equally influential or even that they can be summed in any sensible manner.

Cannabis use was chosen for the initial test because cannabis is the most commonly used drug that is widely illegal, and it is generally acknowledged that cannabis poorly fits standard theories of addiction, including BDMA. The hypothesis was that self-reported cannabis use would be predictable from cannabis constraints, measured by the questionnaire.

## 2. Materials and Methods

### 2.1. Ethics

Participation was on the basis of informed consent, and the research was approved by the ethical procedures of the University of Hull, which conform to the Declaration of Helsinki.

### 2.2. Participants

Participants were recruited using snowball sampling from March 4th to 23rd, 2017; the link to an online questionnaire was shared on Internet forums, and readers were asked to pass the link on to as many other people as possible. In total, 900 participants accessed the survey, but only 323 completed it in full. This high withdrawal rate may have been because participation took place on the Internet, was unsupervised, and unpaid, and the questionnaire was relatively long. Incomplete survey data were discarded. Another 21 cases were removed due to extreme scores and unreliable data, so the final data set included 302 participants. Of these, 67.9% were male (*N* = 205) and 32.1% were female (*N* = 97). Age ranged from 14 to 60 years (mean = 25, SD = 8.0). Participants reported were from 18 different countries, mostly UK (*n* = 172), USA (*n* = 88), Canada (*n* = 12), and Germany (*n* = 8). Between 1 and 3 participants came from Austria, France, Wales, Denmark, Hungary, India, the Netherlands, Chile, Czech Republic, Finland, Greece, Ireland, Italy, Russia, and Sweden. Most reported urban living, in a large city (32.5%), a small city (*n* = 27.5%), or a large town (19.2%). The remainder lived in a small town (13.6%), large village (3.0%), or small village (4.3%). Preliminary data analysis comparing participants from the UK with those from the rest of the world found some significant differences. In the UK, 43.3% of the sample were female, whereas only 29.5% were in the rest of the world (chi^2^ (1d*f*) = 4.695, *p* < 0.05), but place of residence did not differ and age did not differ by the *t* test. Looking at reported days of use in the past 12 months, UK participants were significantly heavier substance users than those from the rest of the world, by *t* tests reporting significantly more frequent use of alcohol, amphetamines, cannabis, cocaine, crack cocaine, ketamine, nitrous oxide, poppers, and tobacco. Consequently, origin (UK or rest of world) was included in the logistic regression analyses below, in case this mediated constraints.

## 3. Materials

### 3.1. Cannabis Constraint Questionnaire

A questionnaire asking about cannabis constraints was developed by the authors based on Hammersley [9]; the questionnaire is shown in Appendix 1. The first section asked participants to rate how much they agreed with a list of reasons why they might control their cannabis use or avoid it all together (constraints), using 5-point Likert scales (strongly disagree, agree, neutral, agree, strongly agree). All constraints were presented with the identical prior statement “*I control my cannabis use, or avoid it all together, because…*” so that participants would not mistakenly rate the statement that comprised the reason rather than the constraint. There were 64 questions in this section divided into 12 sections covering the 15 constraints from Hammersley [9]: (1, 2) Religion and morals; (3) other people; (4, 5) opportunities and other things to do; (6) social factors; (7, 8) does nothing for them and dislike the effects; (9) extent of stress; (10) liking/not liking effects; (11) health; (12) risks of dependence; (13) legal issues; (14) availability; (15) cost. One example question was “*I control my cannabis use, or avoid it all together, because cannabis is too expensive*.” Appendix 1 shows all 64 constraint questions. Some of the sections covered two constraints to make the sections more uniform in length.

### 3.2. Substance Use

The questionnaire next asked for self-report about cannabis and other drug use following Hammersley et al. [21]. This part of the questionnaire asked about estimated frequency of use and self-reported feelings of dependence, covering 23 drugs (some are specific chemicals and some are groups), most of which are illicit: alcohol, amphetamines, benzodiazepines, cannabis, cocaine, crack cocaine, MDMA/Ecstasy, GHB (gamma-hydroxybutyric acid), heroin, ketamine, and novel drugs containing synthetic cannabinoids, Novel drugs that are stimulants (including ones now illegal like mephedrone), LSD (lysergic acid diethylamide), methadone, other opiates, other psychedelics, poppers, psilocybin mushrooms, solvents, steroids, nitrous oxide, tobacco, and “wacks,” which is fictional and only included to indicate unreliable data. Frequency was assessed with the following categories: never; once; 2–5 days (less than once month); 6–12 days (about one a month); 13–24 days (about twice a month); 25–100 days (once or twice a week); 101–365 days (more than twice a week). Quantity data were not collected, due to the complexities of estimating dosage of the psychoactive constituents of cannabis.

### 3.3. Sociodemographic Data

Finally, some basic sociodemographic data were gathered; age in years (as a write-in number), gender, country of residence, and type of place of residence. The questionnaire was converted into an online format using http://www.psytoolkit.com.

### 3.4. Procedure

Participants accessed the online questionnaire via a web link that was posted several times on four Internet fora (reddit.com/r/SampleSize/; reddit.com/r/uktrees/; http://www.reddit.com/r/leaves; reddit.com/r/Drugs/). Each posting was made at approximately 21:00 hours. The posting supplied a link to the questionnaire and read:

“[Survey] Cannabis and reasons for moderating or avoiding use: Could you please complete this survey about reasons for moderating or avoiding cannabis use?”

### 3.5. Data Analysis

Data were analysed using SPSS 22 and SPSS 25. Using survey data with a new instrument, it was not easy to make prior assumptions about the nature and quality of the data or whether it will meet criteria for parametric data analyses. Prior to analysis, the frequency distributions of all variables were checked for normality and skew, considering that substance use measures tend to be positively skewed—most people use infrequently—or bimodal—most people use infrequently, and a large minority use heavily. Two main approaches to data analysis were planned. First, a factor analysis of the 64 cannabis constraint questions and then regression analysis to predict cannabis use frequency from the cannabis constraint questions.

## 4. Results

From the 323 completed surveys, one case was removed due to an extreme score for constraints. A further four cases were removed due to extreme outlying scores for total drug use in the previous 12 months, suggesting either exaggeration or serious polydrug dependence. Another case was removed because the participant reported using the dummy drug “Wacks” suggesting their self-reported data may not be reliable. Finally, 15 cases were removed where age was reported as zero, because this could reflect “skim-responses” where participants give random/unconsidered answers to finish the survey faster. The final data set included 302 participants. Of these, 67.9% were male (*N* = 205) and 32.1% were female (*N* = 97). Age ranged from 14 to 60). The mean age was 25 years (SD = 8.00), with positive skew such that most participants were between 18 and 25.

The constraint questions were theoretically derived, so the first step was to factor analyse these questions to ascertain whether they had a coherent factor structure and, if so, what that structure was and whether it resembled the theoretical constraints proposed by Hammersley [9].

Given that constraint theory has never been tested before, it was decided that confirmatory factor analysis forcing a 15-factor solution would be insufficiently rigorous. Preliminary analyses indicated that (1) not all constraint answers were normally distributed and (2) there were many correlations between constraint questions, so the factor analysis method finally chosen following [22] was exploratory maximum likelihood with oblimin rotation and no limit on the number of factors to be extracted. The model converged after 26 rotations.

Analysing all 64 constraint questions, fifteen factors emerged with eigenvalues >1, which were similar but not identical to the 15 theoretical constraint types.  Table 1 shows how these factors mapped on to the constraints. The new factors are labelled slightly differently to reflect the fact that the items constituting the factors were not identical to those in the constraints. The most important difference was that being concerned about the possibility of addiction emerged as the largest factor and was separate from another smaller factor of actual experiences of addiction. Other main differences were switching to legal highs emerged as a factor; religious or moral beliefs and being jaded regarding consumerism combined to form one factor; only using socially emerged as a factor.

Table 2 is derived from the pattern matrix and shows how each factor loaded on to the questions. Factor scores were saved for use in the logistic regression analyses.

The next stage was to attempt to predict cannabis use in the past year from the factors. Cannabis use in the past 12 months was estimated on a 7-point ordinal scale (never, once, 2–5 times, 6–12 times, 13–24 times, 25–100 times, and101–365 times), and it was distributed bimodally. Therefore, for analysis, it was recoded into two binary variables: nonusers in the year (*n* = 60) vs. users (*n* = 242) and light users (*n* = 135, up to twice a week/25–100 times) vs. heavy users (*n* = 107, more than twice a week/101–365 times) with nonusers excluded. Additionally, participants answered the question whether they thought they were addicted to cannabis (*n* = 44) or not (*n* = 258). These three variables were analysed with three identical logistic regression designs using two blocks. Control variables (gender, alcohol use in the past year, tobacco use in the last year, and country of origin (UK vs. elsewhere) were added in a forwards-entry procedure in block 1. Then, the constraint factors were added in a forward-entry procedure in block 2. Correlated with cannabis use was tobacco use (rho = 0.473, *p* < 0.001) and, less strongly, alcohol use (rho = 0.190, *p*=0.001).

As shown in Tables 2 and 3, those who had used during the past 12 months reported using tobacco and alcohol more often, were less concerned about the possibility of addiction, more likely to use legal highs, and less concerned about illegality and less likely to only use socially when other people used. Gender was significant in block 1 of the regression but not in the final model. This model correctly assigned 93.7% of participants (78.3% of nonusers and 97.5% of users).

Those who had used more than twice a week reported greater addiction concerns, knew more users, reported more availability, fewer unpleasant effects, were less likely to report that it was too expensive, more likely to use for health reasons, and more likely to mention ethical and religious constraints. Gender and tobacco use had been significant in block 1, but were not in the final model. This model correctly assigned 79.3% of participants (79.4% heavy users and 79.3% of light users).

Those who reported that they were addicted to cannabis reported more tobacco use and more addiction concerns and reported they knew more users and they were less concerned about illegality. However, the model was not successful at predicting use as it predicted 100% of nonaddicted users and 0% of addicted users. Gender was significant in block 1 but not in the final model. Of the 44 participants who felt addicted, 39 were in the heavy use group, 3 in the light use group, and 1 in the past use group.

## 5. Discussion

Factor analysis found that in this sample cannabis constraints were structured similarly, but not identically, to the theoretical structure proposed by Hammersley [9]. Concerns about addiction or using too much and experiences of addiction emerged as two separate factors, with the former being the largest factor. Religious or moral beliefs and being jaded regarding consumerism emerged as one small factor. Two factors not in the original theory were using legal highs and only using cannabis socially when other people were using. This factor loaded on to the item “cannabis does nothing for me,” suggesting that some social users experience few effects from the drug.

These constraint factors were able to discriminate well between different levels of cannabis use. Concern about the possibility of addiction, the largest factor, discriminated users who had used in the past 12 months from past users who had not. It also discriminated between current light and heavy users, with light users being more concerned.

Past users also tended to use alcohol and tobacco less, only used when others used and tended to be concerned about legality. They were unlikely to have used legal highs instead of cannabis. This suggests that many past users were cannabis “experimenters” who were never that engaged with it.

Aside from lack of addiction concerns, predictors of heavy use more than twice a week were different. Heavy users knew more users and reported that cannabis was more available, fewer unpleasant effects, were less likely to report that it was too expensive, more likely to use for health reasons, and more likely to mention ethical and religious constraints. All but the last constraint suggest that heavy users tend to be more involved in a cannabis using subculture where it is widely used, available, and affordable; they had fewer constraints on use. Constraint theory predicted that light users would be more likely to mention ethical and religious constraints. Perhaps, this is more salient for users who are already using heavily, which could be construed as “guilt,” something that need not concern light and past users. The minority who felt addicted to cannabis could not be readily discriminated from other users, possibly because the definition and experience of being “addicted” to cannabis is variable and relatively unusual [23]. However, almost all were heavy users. The pattern of constraints discriminating heavy and light users fits with “deviant subculture theory” [24], where engaging with cannabis use requires becoming involved with other users to obtain (or indeed supply) cannabis and to use with. In contrast, light users remained concerned about the possibility of addiction or using too much and were more likely to have experienced unpleasant effects and felt cannabis and people to use it with were less available in terms of access and perceived cost.

These preliminary findings suggest that constraint theory has potential as a means of understanding substance use, and that there is merit in the core idea that people avoid problematic cannabis use by constraining use, rather than being driven towards problematic use by biological and psychological factors beyond their control. In particular, it was not feeling addicted that led to constraining use but being concerned about the possibility of using too much.

### 5.1. Limitations

A criticism of this test of constraint theory of addiction is that perhaps it is obvious that nonusers and lighter users of cannabis will have different attitudes to it than heavier users. There are two defenses to this criticism. First, the questionnaire was not based on arbitrary items approving or disapproving of cannabis but was theoretically derived. By analogy with the much more developed theory of planned behaviour [18, 19], it is not plausible that the results simply reflect a general positive or negative attitude to cannabis. Second, not all the items predicted cannabis in the obvious manner. Notably, there were differences in the constraints that discriminated current year users from nonusers and those that discriminated current year light and heavy users, rather than use and attitudes simply being correlated.

Other limitations of this study include that it was a self-selected volunteer sample and that self-reported use and constraints were collected simultaneously. Ideally, constraints would be studied in a prospective design to predict subsequent use. Another limitation is that the measure of cannabis use was self-report and a relatively crude categorical measure; more sophisticated measures [25] might provide different results.

### 5.2. Implications for Health Promotion

In this volunteer sample, past and light cannabis users were already constraining their use out of concern regarding the potential for addiction, experience of bad effects, and concerns about the legal and employment consequences of use. As cannabis becomes more accepted globally, it will be important not to trivialise its addiction potential for a minority of users, and to continue to emphasise that heavy use (here > twice a week) has greater potential to lead to feelings over overuse or dependence. It is also important to emphasise that if a person experiences adverse effects of use, then they should stop or moderate use, which might involve using less often or using weaker preparations. Finally, breaking the law (where applicable) and facing sanctions at work are also important reasons to constrain use. In short, health promotion advice should be more like that for alcohol. Light and past users are already constrained due to the drug's addiction potential and dire warnings about leading on to harder drugs, or uncontrollable slides to excessive use do not match the cognitions of this sample. As has been noted repeatedly by researchers since the 1970s, e.g., [23, 26], warnings about cannabis risks to lighter or nonusers may be counterproductive because they are not congruent with how they think about cannabis and if use increases may promote complacency about actual risks as these develop. Some light users acutely experience disorientation, psychotic symptoms, or other transient unpleasant effects [23] and some moderate or quit as a result.

Heavy users are less concerned about addiction and unpleasant effects and report fewer constraints on the availability of cannabis and people to use it with. Therefore, a caution is that as cannabis becomes more available legally, there may be an increase in heavy use, as there was historically with cigarettes. However, these data do not inform about whether using regularly of itself is a substantial risk factor for addiction. Nonetheless, it should be noted that 29% (39/135) of heavy users said that they felt addicted. The constraints lacking in heavy use were about availability and perceived cost, which suggests that, to reduce heavy use, people may need to make sustained lifestyle changes rather than simply trying to cutdown whilst having easy access to cannabis and friends to use with.

Overall, these findings suggest that cannabis use is constrained by personal and social choices as well as experiences of unpleasant effects and concerns about addiction. This is not surprising, given that problematic cannabis use is less common than it is for some other drugs and that most cannabis users do not use daily [23]. Furthermore, the question remains as to whether constraints on use of other drugs would be different. It is feasible, but as yet untested, that more problematic drugs would have a different constraint profile.

Currently, health promotion drives for moderate, temperate drinking. As cannabis is becoming more legal around the world and more used as medicine, this message needs to be adopted for cannabis too, although it will be important to emphasise that it should not become a beneficial panacea to be consumed daily ad libitum the way that cigarettes were in their heyday. One advantage of moving away from the BDMA is the possibility of informed discussion and decision by societies about what forms and patterns of substance use are, and are not, ethically and socially acceptable.

## 6. Conclusions

This nonrandom, online, opportunity sample reported cannabis use was constrained primarily by concerns about the potential for addiction and personal and social preferences. Although the present study has some limitations, these findings suggest that constraint theory of addiction has potential as an alternative way of understanding and predicting substance using behavior.

## Figures and Tables

**Table 1:** Factors emerging from factor analysis (maximum likelihood with oblimin rotation) mapped on to theoretical constraints.

Empirical factor	% variance	Theoretical constraint(s)
Addiction concerns	19.8	Recognise immanent dependence
Do not know users	7.7	Lack of friends to use with
Lack of availability	7.0	Lack of availability
Bad effects	5.7	Dislike effects
Disapproval of others	4.2	Family and friends opposed to use
Cannabis too expensive	3.8	Too expensive
Mainly medicinal use	3.3	Health problems
Lack of time/other activities	2.7	Limited opportunities for use
Have alternative ways to relax	2.6	Lack of stress
Switched to legal highs	2.3	New
Ethics and religion	2.1	Religious or moral beliefs/jaded regarding consumerism
Concern about legal consequences	1.9	Legal risks
Other financial priorities	1.9	Other financial priorities
Only use socially	1.8	New
Addiction experiences	1.6	Recognise immanent dependence/health scare or problems/like the drug too much

**Table 2:** Pattern matrix from factor analysis (maximum likelihood with oblimin rotation).

	1	2	3	4	5	6	7	8	9	10	11	12	13	14	15
It is against my family's religion	−00.008	0.110	0.065	−0.012	0.196	−0.007	0.033	0.099	−0.088	0.098	0.326	0.016	−0.018	0.117	0.051
It is against my personal religious beliefs	0.009	0.080	0.073	−0.035	0.087	0.035	0.032	0.075	0.003	0.095	0.499	−0.051	−0.119	−0.002	0.054
It is illegal	0.130	−0.207	0.026	0.017	0.078	0.002	−0.069	0.003	0.009	0.068	0.141	0.520	0.005	−0.043	0.163
Intoxication is morally wrong	0.115	−0.033	−0.007	−0.063	0.016	−0.004	−0.008	−0.042	0.044	0.044	0.696	0.018	−0.055	−0.078	0.083
Consumerist, pleasure-seeking lifestyle	0.039	−0.024	0.077	0.066	−0.017	−0.039	−0.019	−0.074	0.010	−0.002	0.580	0.050	0.013	0.031	−0.017
Worthless dropout lifestyle	0.018	−0.148	−0.102	0.080	0.015	−0.053	−0.168	−0.111	0.189	−0.025	0.534	0.146	0.115	0.019	−0.130
Produced and sold by unscrupulous people	0.002	−0.198	0.087	−0.058	0.161	−0.109	0.035	−0.050	0.109	0.009	0.331	0.167	0.196	−0.029	0.012
Should not pollute one's body	0.112	−0.111	0.093	−0.027	0.068	0.124	−0.021	−0.030	0.175	−0.033	0.526	−0.042	−0.028	0.031	−0.001
Some people disapprove	−0.029	−0.063	−0.106	−0.010	0.493	−0.002	−0.079	−0.071	−0.093	−0.018	0.106	0.355	−0.063	0.118	−0.074
Some people encourage inappropriately	0.046	0.091	−0.228	0.123	0.012	−0.073	0.011	−0.120	0.015	0.077	0.246	0.142	0.040	0.274	−0.179
Family or children disapprove	0.012	0.131	−0.100	−0.027	0.762	−0.003	−0.013	−0.069	−0.002	−0.037	0.021	0.184	−0.079	0.070	0.035
Close friends or partner disapprove	0.105	−0.146	0.058	−0.014	0.577	0.018	−0.031	−0.033	0.044	0.012	−0.030	−0.001	−0.033	0.078	0.016
Family or children disapprove of smoking	0.038	0.110	−0.017	0.002	0.797	−0.036	0.003	−0.037	−0.007	−0.035	0.011	0.124	0.026	−0.014	0.054
Close friends or partner disapprove of smoking	0.069	−0.162	0.087	0.015	0.659	−0.049	0.024	−0.037	0.166	0.007	−0.011	−0.028	0.059	−0.081	−0.033
I do not have time	−0.044	−0.114	0.093	0.103	0.070	−0.034	0.079	−0.680	0.011	0.000	0.041	−0.081	0.020	0.177	0.061
My schedule limits use	−0.030	0.152	0.042	−0.082	0.079	−0.092	0.078	−0.760	−0.205	0.046	−0.123	−0.013	0.029	0.064	0.011
I have important things to do	0.091	−0.004	−0.110	0.110	−0.008	0.044	−0.018	−0.675	0.056	0.015	0.112	0.053	−0.132	−0.114	0.066
It does not fit my lifestyle	0.151	−0.293	−0.009	0.157	0.027	0.075	−0.048	−0.244	0.363	0.047	0.111	0.031	−0.010	0.016	0.122
I do not know many users	0.120	−0.429	0.290	−0.055	0.073	−0.031	−0.055	−0.017	0.062	0.074	−0.089	0.166	0.014	0.087	−0.010
I do not know many people I would enjoy using with	0.165	−0.345	0.276	0.138	−0.001	−0.115	−0.030	−0.085	−0.010	0.058	−0.168	0.228	0.040	0.086	0.058
I want to keep my use private	−0.050	0.053	0.060	−0.082	−0.024	−0.105	0.113	−0.130	−0.093	0.031	−0.015	0.436	−0.063	0.130	−0.196
I mostly use when other people use	0.040	0.019	0.073	−0.001	0.099	−0.043	−0.086	−0.087	0.104	−0.044	0.029	−0.053	−0.047	0.603	−0.075
It does not really affect me	0.031	−0.259	0.133	−0.003	0.066	−0.075	0.029	0.037	0.032	0.023	0.047	−0.027	−0.015	0.311	−0.032
I do not enjoy the effects	−0.073	−0.246	−0.047	0.494	−0.062	0.107	−0.059	0.044	0.347	0.033	0.025	0.093	−0.090	0.111	0.099
I have experienced unpleasant effects	−0.009	0.089	−0.036	0.835	−0.076	0.027	0.008	−0.043	0.060	0.022	−0.094	0.094	−0.010	0.049	−0.008
I have experienced frightening or dangerous effects	0.052	−0.037	0.028	0.790	0.027	−0.044	−0.031	−0.054	−0.047	0.040	0.007	0.069	0.020	−0.078	−0.012
I prefer alcohol or other drugs	0.021	0.121	−0.022	0.218	−0.036	0.090	−0.081	0.004	0.289	0.188	−0.141	−0.043	0.000	0.390	0.170
I am laid back and do not need it	0.058	−0.117	0.115	−0.024	0.028	−0.037	−0.069	−0.110	0.489	0.010	0.034	−0.043	0.052	0.082	0.040
I use it mainly when stressed	−0.002	0.065	0.081	−0.116	−0.077	−0.103	0.487	−0.111	−0.046	0.016	−0.005	0.122	−0.018	0.119	−0.147
I use it mainly for pain	0.008	−0.043	−0.049	−0.038	−0.014	0.059	0.944	−0.075	0.053	0.010	−0.033	0.047	−0.014	−0.038	0.089
I use it mainly for a health condition	0.025	−0.017	−0.028	0.065	0.006	−0.039	0.831	0.028	0.066	−0.033	−0.003	−0.032	0.003	−0.073	0.040
I have found better ways to relax	0.013	0.019	−0.023	0.057	0.026	−0.071	0.083	0.055	0.810	0.071	0.030	−0.005	0.003	0.014	0.007
I have found better ways to have fun	0.042	0.077	0.043	−0.003	0.061	0.010	0.023	0.057	0.833	0.022	0.053	0.007	−0.077	0.111	−0.054
If I am not careful I will use too much	0.089	0.220	−0.010	0.007	0.123	−0.129	−0.008	−0.113	0.015	−0.027	−0.047	0.011	0.026	−0.097	−0.605
It could take over my life	0.427	0.061	−0.007	0.006	0.130	−0.096	−0.130	−0.111	0.097	0.000	0.152	−0.049	0.076	−0.273	−0.351
I appreciate it more if I use less	0.064	0.517	0.129	−0.100	−0.049	−0.101	0.056	−0.224	0.016	−0.020	−0.086	0.102	0.021	0.093	−0.207
I have other things I enjoy doing	0.134	0.095	−0.005	0.077	0.079	0.063	0.057	−0.493	0.214	−0.036	−0.017	0.006	−0.134	−0.106	−0.151
Smoking is bad for my health	0.151	−0.090	0.047	0.025	0.138	0.015	−0.057	−0.015	0.360	0.040	0.129	0.127	−0.053	−0.146	−0.159
Cannabis has damaged my health	0.077	0.073	−0.114	0.209	0.055	−0.079	0.025	−0.087	0.160	0.009	0.101	−0.103	0.120	0.096	−0.390
I have a health problem	0.158	0.023	0.106	0.225	0.083	0.046	0.060	0.067	0.008	0.021	0.249	−0.142	0.053	0.030	−0.136
I have had a bad reaction before	−0.019	−0.007	0.036	0.894	0.006	−0.037	0.010	−0.015	−0.090	0.013	−0.033	−0.048	−0.045	0.002	−0.009
I do not want to become addicted	0.927	0.042	0.002	0.020	0.055	−0.016	0.009	0.021	0.029	−0.024	0.039	0.026	−0.009	0.072	0.084
I think I could become addicted	0.820	−0.017	−0.017	−0.002	0.003	−0.006	0.023	−0.021	−0.071	0.016	−0.015	0.036	−0.038	0.005	−0.139
It leads to other drugs	0.314	−0.053	−0.153	−0.062	−0.044	−0.032	−0.053	0.056	0.139	0.075	0.520	0.076	0.001	0.023	0.090
I use too much and want to cut down	−0.008	0.005	−0.072	−0.027	−0.058	0.040	0.012	−0.008	0.014	0.010	−0.067	0.025	−0.078	0.019	−0.882
I think I am addicted and want to cut down	0.194	−0.036	−0.004	0.032	−0.082	0.007	0.023	0.153	−0.079	0.060	−0.070	−0.014	−0.051	0.091	−0.737
I am worried about getting in trouble with the law	0.003	−0.010	0.117	0.084	0.101	−0.002	0.010	0.052	0.038	0.016	0.011	0.681	0.018	−0.081	−0.015
I have a criminal record	−0.070	−0.053	−0.009	0.042	0.114	0.028	0.116	0.046	−0.017	0.191	0.046	−0.049	−0.005	0.011	−0.163
I am worried about being shamed	0.085	−0.013	0.045	0.042	0.140	0.042	0.010	−0.032	−0.042	0.033	0.012	0.653	−0.035	0.021	−0.016
I would lose my job	0.079	0.109	0.064	0.077	0.183	0.011	0.080	0.070	0.066	0.028	−0.043	0.572	−0.006	−0.053	0.080
I have switched to legal highs because they are legal	0.030	−0.025	0.026	0.115	−0.011	0.006	0.089	−0.001	−0.117	0.596	0.101	−0.007	0.038	0.070	−0.063
I do not know where to get it at the moment	0.033	−0.060	0.809	−0.033	0.015	0.050	−0.081	−0.002	0.029	0.105	0.020	0.035	−0.008	0.048	0.028
I find it difficult to get hold of	0.038	0.064	0.900	0.013	0.032	0.066	−0.019	−0.003	−0.072	0.035	−0.045	0.011	−0.060	0.035	−0.006
I cannot be bothered to get it	−0.030	−0.071	0.629	0.012	−0.016	−0.094	−0.016	−0.063	0.179	−0.012	0.085	0.132	0.002	0.011	0.090
I have to travel too far to get it	−0.083	0.063	0.645	0.054	−0.074	−0.174	0.136	0.021	0.027	−0.013	0.161	0.061	0.044	−0.001	0.014
I have to hang out with people I do not like to get it	0.016	−0.076	0.220	0.024	0.083	−0.180	0.026	−0.037	0.076	0.030	0.103	0.254	0.113	−0.081	0.062
I have switched to legals because they are available	−0.036	0.037	0.113	−0.032	−0.086	0.065	−0.042	−0.082	0.074	0.874	−0.112	0.026	0.037	−0.080	−0.028
It is too expensive	0.007	0.072	0.054	−0.011	0.022	−0.748	−0.054	0.025	0.022	0.055	−0.079	−0.036	−0.235	−0.032	−0.067
Good cannabis is too expensive	0.023	−0.017	−0.037	0.024	−0.020	−0.888	0.086	−0.008	0.028	0.026	−0.047	−0.019	0.038	0.067	0.074
Legals are better value	0.047	−0.017	−0.094	−0.002	−0.002	−0.199	−0.066	0.025	0.072	0.590	0.069	0.050	−0.048	−0.012	0.104
I have other things I want to spend money on	0.085	0.033	0.035	0.082	0.034	−0.103	−0.121	−0.227	0.412	−0.002	0.068	0.043	−0.378	0.043	0.090
I cannot afford cannabis	0.039	−0.067	0.081	0.069	0.131	−0.397	0.080	0.023	−0.059	0.017	0.102	−0.080	−0.445	−0.096	−0.056
I have to spend money on other things	0.010	−0.083	0.010	0.041	−0.034	−0.194	0.040	−0.145	0.091	−0.069	0.098	0.107	−0.662	0.092	−0.096

Factor labels: (1) addiction concerns; (2) do not know users; (3) lack of availability; (4) bad effects; (5) disapproval of others; (6) cannabis too expensive; (7) mainly medicinal use; (8) lack of time/other activities; (9) have alternative ways to relax; (10) switched to legal highs; (11) ethics and religion; (12) concern about legal consequences; (13) other financial priorities; (14) only use socially; (15) addiction experiences.

**Table 3:** Final logistic regression models predicting three levels of cannabis use.

	*B*	S.E.	Wald	d*f*	Sig.	Exp (B)
*Users vs. nonusers*						
Gender	−0.419	0.536	0.611	1	0.434	0.657
Tobacco last 12 months	0.566	0.158	12.780	1	0.000	1.762
Alcohol last 12 months	0.285	0.140	4.105	1	0.043	1.329
Addiction concerns	−1.462	0.316	21.362	1	0.000	0.232
Use legal highs	1.129	0.302	13.945	1	0.000	3.092
Illegality	−0.741	0.296	6.261	1	0.012	0.476
Use when others use	−0.814	0.300	7.379	1	0.007	0.443
Constant	1.186	0.836	2.011	1	0.156	3.274

*Light vs. heavy users*						
Gender	−0.261	0.440	0.353	1	0.553	0.770
Tobacco (last 12 m)	0.091	0.076	1.424	1	0.233	1.095
Addiction concerns	−1.345	0.321	17.619	1	0.000	0.260
Know users	1.427	0.265	29.004	1	0.000	4.168
Lack of availability	−1.071	0.246	18.960	1	0.000	0.343
Unpleasant effects	−0.756	0.266	8.063	1	0.005	0.470
Too expensive	−0.642	0.227	8.025	1	0.005	0.526
Use mainly for health reasons	0.500	0.199	6.318	1	0.012	1.648
Ethics and religion	0.769	0.278	7.641	1	0.006	2.158
Constant	−1.366	0.684	3.993	1	0.046	0.255

*Addicted vs. not*						
Gender	0.597	0.593	1.014	1	0.314	1.817
Tobacco last 12 m	−0.182	0.097	3.556	1	0.059	0.834
Addiction concerns	1.215	0.401	9.194	1	0.002	3.371
Do not know users	−1.804	0.311	33.685	1	0.000	0.165
Illegality	1.231	0.401	9.438	1	0.002	3.423
Constant	3.211	0.925	12.059	1	0.001	24.792

## Data Availability

The data set for this research is available from the corresponding author upon request.

## Supplementary Materials

Supplementary Materials Paper version of the cannabis constraints questionnaire.
