# Supplementary material for: Testing the Constraint Theory of Addiction: Cannabis Constraints Discriminate Users from Nonusers and Heavy from Light Users
Source: J Addict. 2020 May 23;2020:3427270. doi: 10.1155/2020/3427270 (PMC7271244; doi:10.1155/2020/3427270)
Supplement: Supplementary Materials — Paper version of the cannabis constraints questionnaire. [file 3427270.f1.docx]

Appendix 1: Cannabis constraint questionnaire

We want to find out why people control their cannabis use. This is an important question, because most cannabis users do successfully control use. What influences people to do this? By finding out, we hope we will be better able to help prevent drug dependence, and to develop interventions to treat dependence.

The questionnaire will ask about your use of tobacco, alcohol, cannabis and other drugs, because we want to compare your reasons for controlling use with how much you use. The questionnaire is anonymous, so nobody can link this information to you as a person. It also asks some questions about your age, gender, occupation, income and what country you are in.

Thanks very much for your help.

The following questions ask you to rate how much you agree with a long list of reasons why you might control or avoid cannabis use. You can answer these questions whether you have used cannabis or not, and no matter how heavily you are using cannabis at the moment.

*Note: All questions were answered on a 5 point Likert scale:*

*Strongly agree Agree Neutral Disagree Strongly disagree*

*All questions were preceded by the statement:*

*I control my cannabis use, or avoid it all together, because...*

*Finally, the section headings such as MORALS were not printed in the questionnaire given to participants.*

MORALS

It is against my family’s religion

It is against my personal religious beliefs

It is illegal

Intoxication is morally wrong

It is part of a consumerist, pleasure-seeking lifestyle

It is part of a worthless drop-out lifestyle

It is produced and sold by unscrupulous people

One should not pollute one’s body with chemicals

OTHERS

Some people whose opinions matter to me disapprove of my cannabis use

Some people whose opinions matter to me encourage my cannabis use inappropriately

My family and/or children disapprove of cannabis use

My close friends or partner disapproves of cannabis use

My family and/or children disapprove of smoking

My close friends or partner disapproves of smoking

LIFESTYLE

I do not really have the time to use cannabis

My schedule limits when I can use cannabis

I have important things to do and I do not want to be high or suffering the after effects

It does not really fit into my lifestyle

SOCIAL

I do not know many people who use cannabis

I do not know many people that I enjoy using cannabis with

I do not know many people that I am willing to use cannabis with

I want to keep my cannabis use private

I mostly use cannabis because other people are using it

EFFECTS

Cannabis does not really affect me

I do not much enjoy the effects of cannabis

I have experienced effects of cannabis that were unpleasant

I have experienced effects of cannabis that were frightening or dangerous

I prefer the effects of alcohol or other drugs

Write which ones

STRESS

I am a laid-back person and do not really need cannabis

I use cannabis mainly when I am stressed out, so I use less when stress is less

I use cannabis mainly to manage pain, so I use less when not in pain

I use cannabis mainly because I have a specific health condition that it seems to help, so I use less otherwise

I have found better ways to relax

I have found better ways to have fun

LIKE EFFECTS

If I am not careful I spend too much time using cannabis

It could take over my life

I appreciate it more if I do not take it all the time

I also have other things that I enjoy doing which cannabis interferes with

HEALTH

Smoking is bad for my health

I believe that cannabis has damaged my health

I have a health problem that means I should not take cannabis

I have had a bad reaction to cannabis in the past

DEPENDENCE

I do not want to become addicted to cannabis

I think I could easily become addicted to cannabis

I think using cannabis too much leads on to harder drugs

I think I take cannabis far too much and am trying to cut down

I think I am addicted to cannabis and am trying to cut down

LEGAL

I am worried about getting into trouble with the law if I am caught taking cannabis

I have a criminal record and cannot take the risk of being arrested again

I am worried about being shamed or disgraced if I am caught taking cannabis

I would lose my job or get into other serious difficulties if I was caught taking cannabis

I have switched to ‘legal highs’ with cannabis-like effects, because they are legal

AVAILABLITY

I have no idea how to get cannabis at the moment

I often find it difficult to get hold of any cannabis

I cannot be bothered with the trouble I need to go to get cannabis

I have to travel too far to get cannabis

I have to hang out with people I dislike or disapprove of to get cannabis

I have switched to ‘legal highs’ because they are easier to get

COST

Cannabis is too expensive

Good cannabis is too expensive

Legal highs are better value for money

I have other things I would rather spend my money on

I cannot afford cannabis

I have to spend my money on other things

*That is all the questions about cannabis*

We also want to ask you some questions about your use of other substances

| **Which drugs have you used?** | **Tick those you have used**  **below** | **Tick those you have used in the last 4 weeks below** |
| --- | --- | --- |
| Alcohol |  |  |
| Amphetamines (crank, speed, wizz, sulph) |  |  |
| Cannabis (blow, draw, spliffs, hash, grass, ganja) |  |  |
| Cocaine (charlie, coke) |  |  |
| Crack cocaine (rocks, stones) |  |  |
| Ecstasy (MDMA, MDA, ‘E’) |  |  |
| GBH |  |  |
| Heroin (smack, scag, brown, powder, junk, H) |  |  |
| Ketamine (green, special K, ketavet) |  |  |
| Legal highs containing synthetic cannabinoids (including ones now illegal) |  |  |
| Legal highs that are stimulants (including ones now illegal like mephedrone |  |  |
| LSD (acid, trips) |  |  |
| Methadone |  |  |
| Other opiates (such as opium, palfium, codeine) |  |  |
| Poppers (rush, amyl nitrate, butyl nitrate, liquid gold) |  |  |
| Psilocybin mushrooms (magic mushrooms, mushies, ‘shrooms’ liberty cap) |  |  |
| Solvents (such as glues, gases, aerosols, lighter fluid) |  |  |
| Steroids (body-building drugs) |  |  |
| Temazepam (wobblies, mazzies, jellies) |  |  |
| Tobacco (cigarettes) |  |  |
| Valium |  |  |
| Wacks (vids, DHCA, DVDA) |  |  |
| Have you ever injected drugs? |  |  |
| Do you think you are addicted or dependent on tobacco? |  |  |
| Do you think you are addicted or dependent on alcohol? |  |  |
| Do you think you are addicted or dependent on cannabis? |  |  |
| Do you think you are addicted or dependent on any other drug? |  |  |

**The next question asks about how often you have used drugs in the past 12 months. You may not have used a drug at all, or you may have used it in a regular pattern, or irregularly across the year. Circle the number of days that best fits your use, following the suggestions below.**

| Never | Once | 2-5 days  **(less than once month)** | 6-12  days  **(about one a month)** | 13-24 days  **(about twice a month)** | 25-100  days  **(once or twice a week)** | 101-365 days  **(more than twice a week)** |
| --- | --- | --- | --- | --- | --- | --- |

**In the past 12 months, on how many days have you used…**

| Alcohol | Never | Once | 2-5 days | 6-12  days | 13-24 days | 25-100  days | 101-365 days |
| --- | --- | --- | --- | --- | --- | --- | --- |
| Amphetamines (crank, speed, wizz, sulph) | Never | Once | 2-5 days | 6-12  days | 13-24 days | 25-100  days | 101-365 days |
| Cannabis (blow, draw, spliffs, hash, grass, ganja, skunk) | Never | Once | 2-5 days | 6-12  days | 13-24 days | 25-100  days | 101-365 days |
| Products containing synthetic cannabinoids (Spice, XX) | Never | Once | 2-5 days | 6-12  days | 13-24 days | 25-100  days | 101-365 days |
| Cocaine (charlie, coke) | Never | Once | 2-5 days | 6-12  days | 13-24 days | 25-100  days | 101-365 days |
| Crack cocaine (rocks, stones) | Never | Once | 2-5 days | 6-12  days | 13-24 days | 25-100  days | 101-365 days |
| Ecstasy (MDMA, MDA, ‘E’) | Never | Once | 2-5 days | 6-12  days | 13-24 days | 25-100  days | 101-365 days |
| Heroin (H, smack, scag, brown, powder, junk) | Never | Once | 2-5 days | 6-12  days | 13-24 days | 25-100  days | 101-365 days |
| Ketamine (green, special K, ketavet) | Never | Once | 2-5 days | 6-12  days | 13-24 days | 25-100  days | 101-365 days |
| LSD (acid, trips) | Never | Once | 2-5 days | 6-12  days | 13-24 days | 25-100  days | 101-365 days |
| Mephedrone (or other ‘legal high’ type stimulants) | Never | Once | 2-5 days | 6-12  days | 13-24 days | 25-100  days | 101-365 days |
| Methadone | Never | Once | 2-5 days | 6-12  days | 13-24 days | 25-100  days | 101-365 days |
| Other opiates (such as opium, palfium, codeine) | Never | Once | 2-5 days | 6-12  days | 13-24 days | 25-100  days | 101-365 days |
| Poppers (rush, amyl nitrate, butyl nitrate, liquid gold) | Never | Once | 2-5 days | 6-12  days | 13-24 days | 25-100  days | 101-365 days |
| Psilocybin mushrooms (magic mushrooms, mushies, ‘shrooms’, liberty cap) | Never | Once | 2-5 days | 6-12  days | 13-24 days | 25-100  days | 101-365 days |
| Solvents (such as glues, gases, aerosols, lighter fluid) | Never | Once | 2-5 days | 6-12  days | 13-24 days | 25-100  days | 101-365 days |
| Steroids (body-building drugs) | Never | Once | 2-5 days | 6-12  days | 13-24 days | 25-100  days | 101-365 days |
| Temazepam (wobblies, mazzies, jellies) | Never | Once | 2-5 days | 6-12  days | 13-24 days | 25-100  days | 101-365 days |
| Tobacco (cigarettes) | Never | Once | 2-5 days | 6-12  days | 13-24 days | 25-100  days | 101-365 days |
| Valium | Never | Once | 2-5 days | 6-12  days | 13-24 days | 25-100  days | 101-365 days |

Finally, a few questions about you.

What age are you?

What is your gender?

About how much is the income of your household?

What type of place do you live? City, town or large village, small village/ countryside

In what country do you live?
